# Draft Genome Sequences of 28 Actinobacteria of the Family *Microbacteriaceae* Associated with Nematode-Infected Plants

**DOI:** 10.1128/MRA.01400-20

**Published:** 2021-03-04

**Authors:** Sergey V. Tarlachkov, Irina P. Starodumova, Lubov V. Dorofeeva, Natalia V. Prisyazhnaya, Tatiana V. Roubtsova, Vladimir N. Chizhov, Steven A. Nadler, Sergei A. Subbotin, Lyudmila I. Evtushenko

**Affiliations:** a All-Russian Collection of Microorganisms (VKM), G. K. Skryabin Institute of Biochemistry and Physiology of Microorganisms, Pushchino Scientific Center for Biological Research of the Russian Academy of Sciences, Pushchino, Russia; b Department of Plant Pathology, University of California, Davis, California, USA; c Center of Parasitology of A. N. Severtsov Institute of Ecology and Evolution of the Russian Academy of Sciences, Moscow, Russia; d Department of Entomology and Nematology, University of California, Davis, California, USA; e California Department of Food and Agriculture, Sacramento, California, USA; University of Maryland School of Medicine

## Abstract

Draft genome sequences of 28 strains of *Microbacteriaceae* from plants infested by plant-parasitic nematodes were obtained using Illumina technology. The sequence data will provide useful baseline information for the development of comparative genomics and systematics of *Microbacteriaceae* and facilitate understanding of molecular mechanisms involved in interactions between plants and nematode-associated bacterial complexes.

## ANNOUNCEMENT

Members of the family *Microbacteriaceae* (class *Actinobacteria*) inhabit various terrestrial and aquatic ecosystems and often occur in plants as endophytes and pathogens (1, 2). Plant-pathogenic species of the genus *Rathayibacter* belonging to this family are transmitted to their host plants by gall-forming nematodes of the genus *Anguina* (Anguinidae) (3, 4). Along with *Rathayibacter*, some other *Microbacteriaceae*, including members of the genera *Agreia*, *Leifsonia*, *Microbacterium*, and *Plantibacter*, were recovered from plant galls induced by Anguinidae and from leaf tissues of Tanacetum vulgare infested by Aphelenchoides fragariae (1, 5–8).

Novel nematode-associated strains of *Microbacteriaceae* were isolated from plant galls induced by different anguinids and from plant tissues affected by *Aphelenchoides* species (Table 1). The air-dried plant samples were soaked in distilled water for 1 h, washed twice with sterile distilled water, placed in 0.85% NaCl solution, and milled. One drop of the obtained suspension was plated onto modified *Corynebacterium* agar (9) or Reasoner’s 2A (R2A) agar (Fluka Analytical, USA) and incubated for 1 to 3 weeks at room temperature (18 to 24°C). The isolated strains were identified on the basis of matrix-assisted laser desorption ionization (MALDI) mass spectra and 16S rRNA gene sequences as described previously (10, 11) and deposited in the All-Russian Collection of Microorganisms (VKM; http://www.vkm.ru).

**TABLE 1:** Characteristics and DDBJ/ENA/GenBank accession numbers of genome sequences

Organism	Plant	Nematode	Geography	No. of reads	Coverage (×)	No. of scaffolds	Scaffold *N*_50_ (bp)	Genome size (Mbp)	G+C content (%)	No. of proteins	SRA accession no.	GenBank accession no.
Agreia pratensis VKM Ac-2874	Poa annua	Anguina pacificae	San Francisco, CA, USA	7,950,938	304	13	1,078,817	3.8	65.2	3,532	SRR13176583	JADKRN000000000
Clavibacter michiganensis subsp. *michiganensis* VKM Ac-1790	*Agrostis* sp.	Anguina agrostis	Sakhalin Island, Russia	16,949,770	737	9	808,662	3.3	72.7	3,036	SRR13176582	JADKRO000000000
Clavibacter michiganensis subsp. *phaseoli* VKM Ac-2886	Sambucus racemosa	Aphelenchoides ritzemabosi	Moscow Region, Russia	18,464,076	785	21	2,249,679	3.4	73.2	3,186	SRR13176571	JADKRP000000000
*Clavibacter* sp. strain VKM Ac-2542	Elymus repens	Anguina agropyri	Moscow Region, Russia	15,327,488	656	5	1,206,839	3.3	73.0	3,103	SRR13176562	JADKRQ000000000
*Clavibacter* sp. strain VKM Ac-2872	*Poa annua*	*Anguina pacificae*	San Francisco, CA, USA	16,543,808	675	12	2,167,218	3.5	72.7	3,276	SRR13176561	JADKRR000000000
*Clavibacter* sp. strain VKM Ac-2873	Agrostis capillaris	*Anguina agrostis*	Washington, USA	17,375,186	751	14	534,200	3.3	73.2	3,064	SRR13176560	JADKRS000000000
Curtobacterium flaccumfaciens VKM Ac-1386	*Agrostis* sp.	*Anguina agrostis*	Iturup Island, Russia	12,378,592	457	18	376,811	3.9	70.9	3,687	SRR13176559	JADKRT000000000
Curtobacterium flaccumfaciens VKM Ac-1795	*Agrostis* sp.	*Anguina agrostis*	Sakhalin Island, Russia	11,498,758	417	36	428,105	3.9	70.7	3,692	SRR13176558	JADKRU000000000
*Curtobacterium* sp. strain VKM Ac-1376	*Poa annua*	Subanguina radicicola	Moscow Region, Russia	9,503,968	362	61	195,312	3.8	70.4	3,507	SRR13176557	JADKRV000000000
*Curtobacterium* sp. strain VKM Ac-1393	*Calamagrostis* sp.	Heteroanguina graminophila	Kunashir Island, Russia	12,098,310	438	20	491,012	3.9	70.5	3,681	SRR13176556	JADKRW000000000
*Curtobacterium* sp. strain VKM Ac-1395	Festuca rubra	Anguina graminis	Moscow Region, Russia	9,679,080	355	18	628,495	3.9	71.1	3,670	SRR13176581	JADKRX000000000
*Curtobacterium* sp. strain VKM Ac-1796	*Centaurea* sp.	Mesoanguina picridis	North Caucasus, Russia	12,573,324	503	9	630,474	3.6	71.0	3,364	SRR13176580	JADKRY000000000
*Curtobacterium* sp. strain VKM Ac-2865	*Agrostis capillaris*	*Anguina agrostis*	Washington, USA	13,029,810	500	15	428,310	3.7	71.4	3,461	SRR13176579	JADKRZ000000000
*Curtobacterium* sp. strain VKM Ac-2884	*Agrostis capillaris*	*Anguina agrostis*	Washington, USA	9,597,106	354	15	694,950	3.9	70.9	3,646	SRR13176578	JADKSA000000000
*Curtobacterium* sp. strain VKM Ac-2887	*Agrostis capillaris*	*Anguina agrostis*	Moscow Region, Russia	12,452,164	427	57	242,003	4.2	70.6	3,952	SRR13176577	JADKSB000000000
*Curtobacterium* sp. strain VKM Ac-2889	*Agrostis* sp.	*Anguina agrostis*	Kunashir Island, Russia	11,453,674	457	9	940,853	3.6	71.0	3,357	SRR13176576	JADKSC000000000
*Frigoribacterium* sp. strain VKM Ac-1396	Festuca rubra	*Anguina graminis*	Moscow Region, Russia	11,568,590	486	8	1,115,953	3.4	71.7	3,088	SRR13176575	JADKSD000000000
*Frigoribacterium* sp. strain VKM Ac-2530	Tanacetum vulgare	Aphelenchoides fragariae	Moscow Region, Russia	19,836,426	834	5	1,902,771	3.4	72.7	3,110	SRR13176574	JADKSE000000000
*Frondihabitans* sp. strain VKM Ac-2883	*Fagus* sp.	*Litylenchus* sp.	New York State, USA	6,461,938	253	24	417,102	3.7	67.3	3,391	SRR13176573	JADKSF000000000
*Herbiconiux* sp. strain VKM Ac-1786	*Elymus repens*	*Anguina agropyri*	Moscow Region, Russia	16,964,600	622	4	2,263,567	3.9	71.1	3,649	SRR13176572	JADKSG000000000
*Microbacterium* sp. strain VKM Ac-2870	*Poa annua*	Anguina pacificae	San Francisco, CA, USA	11,489,968	526	12	704,918	3.1	68.3	2,905	SRR13176570	JADKSH000000000
*Plantibacter* sp. strain VKM Ac-2876	*Poa annua*	*Anguina pacificae*	San Francisco, CA, USA	9,413,878	338	7	1,281,621	4.0	69.5	3,702	SRR13176569	JADKSI000000000
*Plantibacter* sp. strain VKM Ac-2880	Klasea latifolia	Mesoanguina picridis	Iran	11,839,552	422	7	2,540,737	4.0	69.4	3,765	SRR13176568	JADKSJ000000000
*Plantibacter* sp. strain VKM Ac-2885	Festuca rubra	*Anguina graminis*	Moscow Region, Russia	12,004,120	410	4	2,630,716	4.2	69.2	3,853	SRR13176567	JADKSK000000000
*Pseudoclavibacter* sp. strain VKM Ac-2867	*Agrostis capillaris*	*Anguina agrostis*	Washington, USA	10,413,762	357	47	195,216	4.2	68.2	3,812	SRR13176566	JADKSL000000000
*Pseudoclavibacter* sp. strain VKM Ac-2888	*Tanacetum vulgare*	*Aphelenchoides fragariae*	Moscow Region, Russia	11,942,026	409	7	3,268,120	4.2	68.5	3,851	SRR13176565	JADKSM000000000
*Rathayibacter* sp. strain VKM Ac-2878	Danthonia californica	Anguina danthoniae	Fort Ross, CA, USA	11,690,574	569	9	860,400	2.9	69.9	2,692	SRR13176564	JADKSN000000000
*Rathayibacter* sp. strain VKM Ac-2879	*Danthonia californica*	*Anguina danthoniae*	Fort Ross, CA, USA	9,176,840	448	9	860,400	2.9	69.9	2,690	SRR13176563	JADKSO000000000

For genome sequencing, DNA was extracted with a QIAamp DNA minikit (Qiagen, Germany) from biomass grown in liquid peptone-yeast medium as described previously (7) or using cells incubated on R2A agar for 2 to 3 days at 28°C. DNA library construction and sequencing were conducted by Novogene Co., Ltd., using a NEBNext Ultra II DNA library prep kit for Illumina (New England Biolabs) following the manufacturer’s recommendations. Pooled DNA libraries were sequenced on an Illumina NovaSeq 6000 instrument to obtain 150-bp paired-end reads.

Default parameters were used for all software unless otherwise specified. The quality of the reads was checked with FastQC 0.11.8 (12). Adapter sequences and low-quality regions in raw reads were cut with Trimmomatic 0.39 (13) with the following options: ILLUMINACLIP:TruSeq3-PE-2.fa:2:30:10; SLIDINGWINDOW:4:20; MINLEN:50. Trimmed reads were assembled using SPAdes 3.14.1 (14) with the following options: --cov-cutoff, auto, and --isolate. The quality of assembly was assessed with QUAST 5.0.2 (15). Assemblies were annotated with NCBI PGAP (16) and the RAST Web server (17, 18).

Additional comparative phenotypic study of the sequenced strains, along with genome-wide analyses of phylogenetically closely related plant endophytes and pathogens, will facilitate understanding of their role in bacterial-nematode complexes, including mechanisms of molecular interactions between members of these complexes and plants.

### Data availability.

These whole-genome shotgun projects have been deposited in DDBJ/ENA/GenBank under the accession numbers listed in Table 1.
